# A pseudo-softmax function for hardware-based high speed image classification

**DOI:** 10.1038/s41598-021-94691-7

**Published:** 2021-07-28

**Authors:** Gian Carlo Cardarilli, Luca Di Nunzio, Rocco Fazzolari, Daniele Giardino, Alberto Nannarelli, Marco Re, Sergio Spanò

**Affiliations:** 1 Department of Electronic Engineering, University of Rome “Tor Vergata”, 00133 Rome, Italy; 2 Department of Applied Mathematics and Computer Science, Danmarks Tekniske Universitet, 2800 Kongens Lyngby, Denmark

**Keywords:** Electrical and electronic engineering, Computer science

## Abstract

In this work a novel architecture, named pseudo-softmax, to compute an approximated form of the softmax function is presented. This architecture can be fruitfully used in the last layer of Neural Networks and Convolutional Neural Networks for classification tasks, and in Reinforcement Learning hardware accelerators to compute the Boltzmann action-selection policy. The proposed pseudo-softmax design, intended for efficient hardware implementation, exploits the typical integer quantization of hardware-based Neural Networks obtaining an accurate approximation of the result. In the paper, a detailed description of the architecture is given and an extensive analysis of the approximation error is performed by using both custom stimuli and real-world Convolutional Neural Networks inputs. The implementation results, based on CMOS standard-cell technology, compared to state-of-the-art architectures show reduced approximation errors.

## Introduction

The softmax function is one of the most important operators in the field of Machine Learning^1^. It is used in the last layer in classification Neural Networks (NN) and also in Convolutional Neural Networks (CNN) to normalize the raw output of such systems.

**Figure 1:**
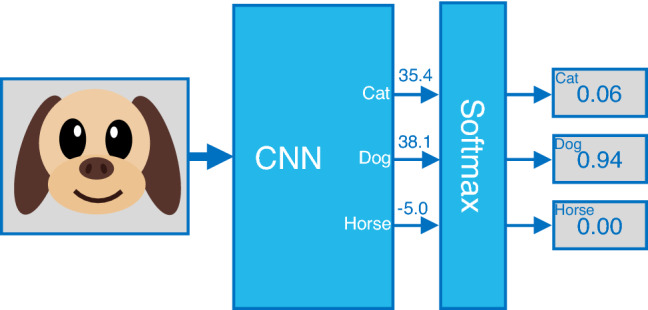
Example of a three classes CNN: Cats, Dogs, and Horses.

The softmax function equation is:

1 $$\begin{aligned} p_i=\frac{e^{x_i}}{\sum _{k=1}^{N} e^{x_k}} \end{aligned}$$

where $x_i$ are the outputs of a machine learning network and $i=,1, \ldots , N$. In other words, the outputs of the network $x_i$ are processed to represent the probability of the inference output $p_i$ to belong to a certain class (Fig. 1).

In recent years, the literature proposed many hardware architectures for the inference process of NNs and CNNs both on ASIC and FPGA^2–4^, characterized by high speed and low power consumption. The optimization in the hardware architectures is obtained both by the use of approximation algorithms and by the integer quantization of the arithmetic, usually by using 8 bits integers (INT8).

Unfortunately, the softmax function, unlike other operators used in Machine Learning^5–8^, cannot be easily implemented because of the exponential and division operators. Moreover, even off-the-shelf NN and CNN synthesis tools are not able to provide a hardware softmax implementation^9,10^, and the function is computed by using a standard software approach.

In this work, we introduce the pseudo-softmax architecture with the aim to allow for an efficient hardware implementation of the softmax layer in hardware implemented NNs and CNNs.

Different solutions for the hardware implementation of the softmax function can be found in the literature but, unfortunately, each work focuses on different aspects of the design process, making comparisons not too easy.

In the following, recent relevant work on the softmax function is summarized by highlighting the most innovative aspects of each work.

Yuan^11^ proposed an implementation that uses a logarithmic transformation to avoid the division, but no final conversion to the original domain is considered. The exponential operations are simply carried out via Look Up Tables (LUT). A comparison on the number of operations performed by a standard LUT-based divisor and his proposed method is given.

Geng et al.^12^ proposed two architectures that compute the exponential function both via LUT and linear approximation. The division is carried out by finding the closest power of 2, thus only shift operations are needed. The accuracy was tested on real CNNs and an ASIC implementation is presented.

Li et al.^13^ proved that LUT implementations for the exponential function are the best trade-off between precision and speed, if compared to Taylor’s series and CORDIC^14^ implementations. The division is performed by bit-shifting. They presented two serial implementations both in FPGA and ASIC giving data about clock speed and resources. No information is provided on the latency of the architecture.

Baptista et al.^15^ proposed a High Level Synthesis (HLS) FPGA implementation for a specific CNN application. The exponents of the softmax formula are split into integer and fractional parts. The integer parts are evaluated by using a ROM approach, while polynomial approximation is used for the fractional parts. The results are given as the global accuracy of the overall Machine Learning system, not focusing on the softmax computation.

Wang et al.^16^ proposed an interesting architecture that exploits the fact that every number can be split in integer and fractional part. The implementation avoids any straightforward division or exponential operation by using Leading One Detectors (LOD), bit shifters, and constant multipliers. The authors considered the output of their system correct if the difference with respect to the original softmax value lies below a given threshold. The architecture was implemented both on ASIC and FPGA and information about the clock frequency, hardware resources, power dissipation, and throughput are provided.

Sun et al.^17^ proposed a FPGA serial implementation that splits every exponential operation in more operations to reduce the size of each ROM. The division is carried out via bit-shifting. The authors provided information about the clock frequency, hardware resources, and power dissipation, but no data about the precision of the system is provided.

Hu et al.^18^ proposed their Integral Stochastic Computation (ISC) to evaluate the exponent operator. The division is avoided by a logarithmic transformation. No data about the precision of the system is provided. They implemented the architecture by using an FPGA but the maximum achievable clock frequency is not provided.

Du et al.^19^ proposed a tunable precision block for the exponentiation based on a variable number of LUTs. The architecture has been implemented both in ASIC and FPGA and data about clock frequency, hardware resources, and power dissipation are provided.

Kouretas and Paliouras^20^ implemented an approximated equation that takes into account only the exponents of the softmax formula and that replaces the summation with the highest input value. They compute the accuracy by using custom inputs and they show the hardware resources needed for an ASIC implementation of the architecture.

Wang et al.^21^ showed a CNN application that makes use of software-tunable softmax layer in terms of precision, but no detailed data about the softmax implementation is provided.

Di Franco et al.^22^ proposed a straightforward FPGA implementation of the softmax by using a linear interpolating LUT for the exponential function. No information about the accuracy is provided.

Kouretas and Paliouras^23^ extended their previous work^20^ by improving the accuracy analysis by using real-world CNNs and by adding information about the ASIC implementation.

At the time of the writing, the work in^23^ can be considered the state-of-the-art in hardware implementations of the softmax function, and it will be used for comparisons in the following sections.

## Pseudo-softmax function

In order to simplify the computation of the softmax function in Eq. (1), we introduce a new approximated expression named *pseudo-softmax*:

2 $$\begin{aligned} \widetilde{p}_i=\frac{2^{x_i}}{\sum _{k=1}^{N} 2^{x_k}} \end{aligned}$$

in which the exponent base *e* is replaced by 2. An extensive analysis of the error introduced is discussed in the following section. As in the case of the softmax function, the summation of the pseudo-softmax outputs is always equal to one. Consequently, the values $\widetilde{p}_i$ can be interpreted as probabilities.

As stated in the Introduction, the hardware implementations of NN systems make use of the integer quantization, typically 8-bit integers (INT8). The reason of using powers of two $2^{x_i}$ in Eq. (2) is that the integer numbers $x_i$ can be interpreted as the exponent of floating-point (FLP) numbers, allowing for an efficient hardware implementation.

According to the conventional base-2 FLP representation, a positive number *a* is represented as:

3 $$\begin{aligned} a=2^b \cdot 1 \cdot c, \end{aligned}$$

where *b* is the integer exponent and *c* is the fractional mantissa. Consequently, the numerator in Eq. (2) can be considered as a number $a_i=2^{x_i}$, where $b=x_i$ and $c=0$, and Eq. (2) can be rewritten as

4 $$\begin{aligned} \widetilde{p}_i=\frac{a_i}{\sum _{k=1}^{N} a_k}. \end{aligned}$$

Similarly, for the denominator in Eq. (4), the sum can be rewritten as

$$\begin{aligned} {sum}=\sum _{k=1}^{N} {a_k}=2^{\text {exp}_{\text {sum}}} \cdot 1 \cdot \text {mant}_{\text {sum}}, \end{aligned}$$

and the pseudo-softmax function as

5 $$\begin{aligned} \widetilde{p}_i=\frac{a_i}{2^{\text {exp}_{\text {sum}}} \cdot 1 \cdot \text {mant}_{\text {sum}}} . \end{aligned}$$

Substituting back $a_i=2^{x_i}$ in Eq. (5), we obtain

6 $$\begin{aligned} \widetilde{p}_i=\frac{2^{x_i}}{2^{\text {exp}_{\text {sum}}}} \cdot \frac{1}{ 1 \cdot \text {mant}_{{sum}}} . \end{aligned}$$

that can be rewritten as

7 $$\begin{aligned} \widetilde{p}_i=2^{x_i-\text {exp}_{\text {sum}}} \cdot \frac{1}{ 1 \cdot \text {mant}_{\text {sum}}}. \end{aligned}$$

The expression Eq. (7) of the pseudo-softmax function shows that the output $\widetilde{p}_i$ is a FLP number with exponent $(x_i-\text {exp}_{\text {sum}})$, and with mantissa $1/(1 \cdot \text {mant}_{\text {sum}})$, i.e., the reciprocal of the mantissa of the summation. The mantissa is common (constant) for all $\widetilde{p}_i$s, and it is only computed once.

### Hardware architecture

**Figure 2:**
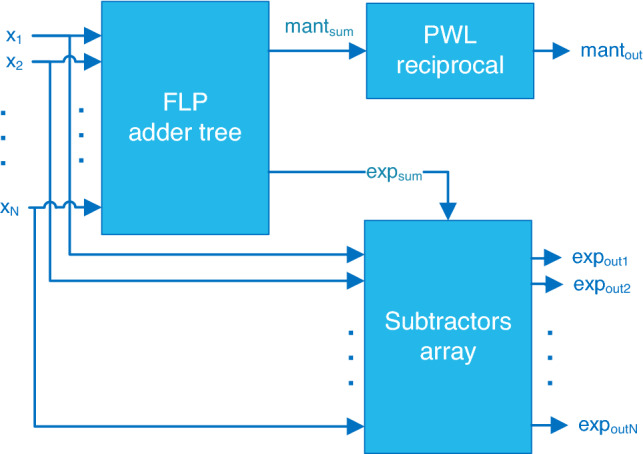
Pseudo-softmax function top level architecture.

The pseudo-softmax function in Eq. (7) is implemented by using the hardware architecture shown in Fig. 2.

As stated in the Introduction, the 8-bit integers inputs $x_i$ with range $[-128,127]$ are interpreted as the exponents of FLP numbers. The denominator of Eq. (7) is the mantissa of the FLP number *sum*. The outputs are unsigned FLP numbers

8 $$\begin{aligned} \widetilde{p}_i=2^{\text {exp}_{\text {out}i}} \cdot { 1 \cdot \text {mant}_{\text {out}}}. \end{aligned}$$

represented by using 17 bits: 9-bit exponent, and 8-bit fractional mantissa with implicit integer bit (always 1). The 9-bit exponent (unbiased) guarantees for overflows for maximum values $x_i=127$ and number of inputs $N<128$. There is no representation for zero, that can be determined by comparing $\widetilde{p}_i$ to a sufficiently small threshold value. The negative exponent makes the floating-point number smaller than 1.0, but all output numbers are positive. Therefore, the sign bit is not necessary.

The unit in Fig. 2 is composed of three main blocks: a tree of FLP adders to compute $sum=2^{\text {exp}_{\text {sum}}} \cdot 1 \cdot \text {mant}_{\text {sum}}$; a piece-wise linear (PWL) interpolation block to compute the reciprocal, and an array of integers subtractors computing $(x_i-\text {exp}_{\text {sum}})$.

In the following subsections, more detail on the main blocks in Fig. 2 is given.

The wordlenght sizes in the circuits are represented in gray characters, thin arrows represent 1-bit signals.

#### Floating-point adder tree

We opted for a binary tree of FLP adders, that is modular and easy to design. If delay (for throughput) is problematic, the binary tree can be easily pipelined, after each adder, to meet the timing constraints.

**Figure 3:**
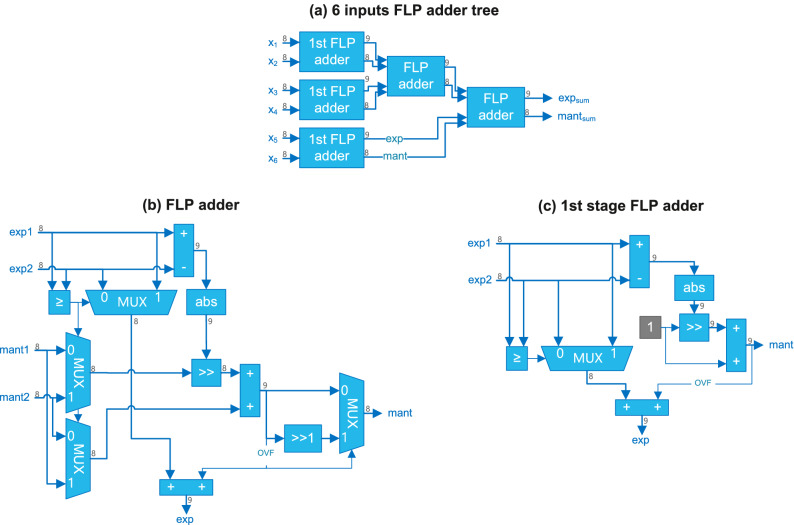
(**a**) Example of 6 8-bit inputs FLP adder tree. (**b**) Architecture of a FLP adder. (**c**) Architecture of the optimized FLP adder used in the first level of the tree.

The architecture of the FLP adder tree for N = 6 is shown in Fig. 3a.

The $x_i$ of Eq. (2) are the exponents of FLP numbers and their mantissas is 1.0.

The architecture of the FLP adder^24^ is shown in Fig. 3b. Since it operates on positive FLP numbers, its architecture is simplified.

First, the exponents difference *d* is computed to find the amount of shifting necessary for the alignment of the mantissas. The largest exponent is selected as the exponent of the result. The alignment is performed by a barrel shifter (block $\gg$ in Fig. 3b) by shifting *d* positions to the right the mantissa of the smallest number. If $d\ge 8$, the mantissa of the smallest number is flushed to zero, and no actual addition is performed. When $d=0$, same exponent, the addition of the normalized mantissas results in an overflow ($\text{ mant } \ge 2.0$) and the result must be normalized by incrementing by one the exponent, and by dividing the mantissa by two, i.e., right-shifting the result 1 position (block $\gg 1$).

An additional simplification is done for the FLP adder in the first level of the adder tree (Fig. 3c). Since, the input values $x_i$ are power of two’s numbers and their mantissas is 1.0, there is no need to swap the mantissas (identical) according to *d*. The barrel shifter is also simplified because its input is the constant 1.0. When $d=0$, i.e., $x_i=x_j$, the result of the addition of the two mantissas is $\text{ mant }=2.0$ (overflow). However, since the fractional bits are all zero, right-shifting is not necessary, and the normalization is done by incrementing the exponent of the result only.

#### Piece-wise linear reciprocal block

In the computation of the probabilities $\widetilde{p}_i$, the mantissa is common to all $\widetilde{p}_i$s, and consequently, a single reciprocal operation is sufficient. Moreover, because of the normalization, the mantissa is in the range [1, 2).

For the reciprocal $y = 1/x$, we opted for a piece-wise linear (PWL) polynomial approximation in two intervals

9 $$\begin{aligned} \tilde{y}= \left\{ \begin{array}{ll} 1.59375-0.625 \cdot x &{} \text {if } x < 1.5 \\ 1.125-0.3125 \cdot x &{} \text {if }x \ge 1.5 \\ \end{array} \right. . \end{aligned}$$

The coefficients of the polynomials were chosen, by incremental refinements, as the closest to powers of two to simplify the hardware. By expressing in binary the coefficients of (9) and as powers of two, we have

10 $$\begin{aligned} \tilde{y}= \left\{ \begin{array}{llll} 1.10011|_2 &{}-&{} x (2^{-1}+ 2^{-3}) &{} \quad \text {if } x < 1.5 \\ 1.00100|_2 &{}-&{} x (2^{-2}+2^{-4}) &{} \quad \text {if }x \ge 1.5 \\ \end{array} \right. . \end{aligned}$$

**Figure 4:**
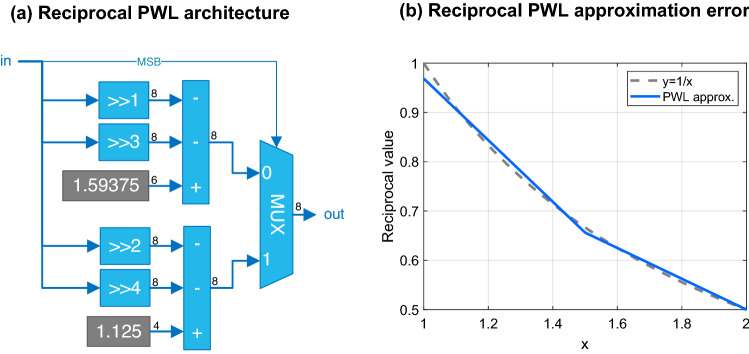
(**a**) Architecture of the PWL reciprocal block. (**b**) PWL reciprocal function for *x* in the range [1,2).

The resulting reciprocal approximation unit is shown Fig. 4a.

Since the intervals in Eq. (10) are determined by $\text {mant}_{\text {sum}}$ being greater or smaller than 1.5, the MSB of the fractional part of the mantissa, bit with weight $2^{-1}$, is used to select the interpolating polynomial.

Figure 4b shows the plots of $y=1/x$ and of the interpolating polynomials in [1.0, 2.0).

The reciprocal approximation error is obtained by exhaustive simulation in fixed-point. The maximum absolute error is $0.03125< 2^{-5}$ obtained for $x=1.0$ (Fig. 4b), while the average error is $0.011151 < 2^{-7}$. This is a good trade-off between error and hardware complexity (Fig. 4a).

#### Pseudo-Boltzmann architecture for reinforcement learning hardware accelerators

The proposed pseudo-softmax formula in Eq. (2) can be adapted to implement the Boltzmann action selection policy^25^ for Reinforcement Learning (RL) systems. The design of an efficient architecture would allow for such policy to be implemented in the state of the art of RL hardware accelerators^26,27^.

The formula of the Boltzmann policy is:

11 $$\begin{aligned} B_i=\frac{e^{{x_i}/\tau }}{\sum _{k=1}^{N} e^{{x_k}/\tau }} . \end{aligned}$$

It is straightforward to see that Eq. (1) is a special case of Eq. (11) where the temperature coefficient $\tau =1$ (Kelvin). In order to avoid the division operation by $\tau$, we can consider a power of two approximation $\tau =2^T$ obtaining the pseudo-Boltzmann equation:

12 $$\begin{aligned} \widetilde{B}_i=\frac{2^{x_i/2^T}}{\sum _{k=1}^{N} 2^{x_k/2^T}} . \end{aligned}$$

The corresponding hardware architecture is obtained with minor modifications of the pseudo-softmax architecture shown Fig. 2.

## Results

In this section we provide extensive testings to analyze the precision of the proposed pseudo-softmax. An analysis on the quantization of the architecture is also provided. The Psedo-Softmax operator is compared to the hardware-based softmax design illutsrated in^23^. Then, we show the pseudo-softmax ASIC implementation results based on a 90 nm standard-cell CMOS technology. The results are given in terms of propagation delay, silicon area and power dissipation. Our results are compared with the implementation in^23^.

### Approximation error analysis

In order to validate the pseudo-softmax function, we performed extensive simulations using both custom inputs and real data from Convolutional Neural Networks (CNN). We also compared our results with those obtained by the hardware-based softmax implementation described in^23^.

To easily compare the simulation results with those of^23^, the performance is evaluated by computing the Mean Square Error (MSE)

13 $$\begin{aligned} \text {MSE}= \frac{1}{n} \sum {\left( y - \widetilde{y} \right) ^2} . \end{aligned}$$

Moreover, to be consistent with^23^, the tests were performed in floating-point by assuming quantized integer inputs.

#### Custom generated inputs

One test consisted in applying **random uniformly distributed inputs** in the range $[-2^{-7},2^7-1]$ (INT8) to the pseudo-softmax module. The number of inputs *N* tested was in the range $N=$[2,1000], being *N* the number of classes of a neural network. For every chosen *N*, we applied 10,000 random patterns to the system’s inputs $x_i$.

**Figure 5:**
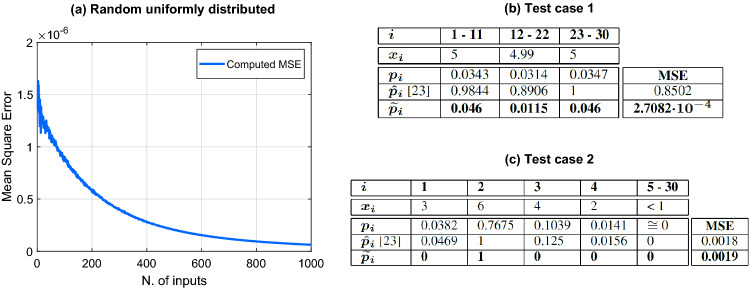
(**a**) MSE of random uniformly distributed inputs vs number of inputs. (**b**) Inputs and outputs for test case n. 1; MSE comparison between^23^ and proposed architecture. (**c**) Inputs and outputs for test case n. 2; MSE comparison between^23^ and proposed architecture.

The MSE as a function of the number of inputs *N*, is shown in Fig. 5a.

The plot shows that the MSE decreases as the number of inputs increases.

We now compare our pseudo-softmax function to the design shown in^23^ by using the same network parameters: 10-bit inputs an $N=30$. The input values in^23^ are chosen in such a way to push the softmax hardware in appropriate “corner cases”.

For the **first test case**, the input values $x_i$ are close to 5. These are shown in the second row of Figure 5b. The other rows in Fig. 5b display the values $p_i$ for the softmax function, $\hat{p}_i$ for the softmax approximation of^23^, and $\widetilde{p}_i$ for the pseudo-softmax.

Since the input values $x_i$ in Fig. 5b are close to each other, also the softmax values $p_i$ are rather close. The outputs $\hat{p}_i$ are much larger and their sum is larger than 1, violating the principle that the softmax is a probability density function (PDF). In contrast, the outputs $\widetilde{p}_i$, preserve the features of PDFs and all values are close to $p_i$.

The MSE value for $\widetilde{p}_i$ is $\text {MSE}_{\widetilde{p}_i} = 2.7082 \times 10^{-4}$, while $\text {MSE}_{\hat{p}_i} = 0.8502$.

Figure 5c reports the results of the **second test case** using the same organization of Fig. 5b.

In this case, the first four inputs $x_i$ are significantly larger than the remaining $x_i < 1$. Also in this case, the sum of $\hat{p}_i$ outputs is larger than 1, while the $\widetilde{p}_i$ mantains a PDF behavior. However, the MSE of the approximation are almost the same, since $\text {MSE}_{\widetilde{p}_i} = 0.0019$ and $\text {MSE}_{\hat{p}_i} = 0.0018$.

#### Convolutional Neural Networks benchmarks

The performance of the pseudo-softmax approximation algorithm is also evaluated with real data using the set of tests performed in^23^ based on standard CNNs.

The test is based in the ImageNet dataset^28^ consisting in classifying 1000 images. The test is performed by 10,000 inferences on the following networks:

1. ResNet-50^29^,
2. VGG-16^30^,
3. VGG-19^30^,
4. InceptionV3^31^,
5. MobileNetV2^32^.

**Figure 6:**
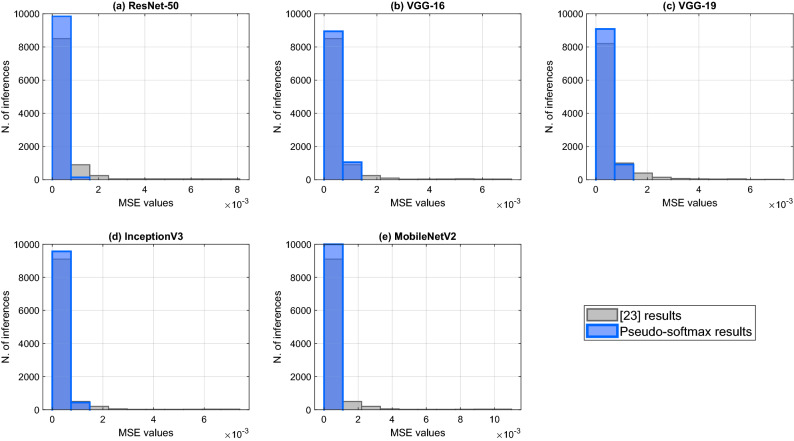
(**a**) ResNet-50, (**b**) VGG-16, (**c**) VGG-19, (**d**) InceptionV3, (**e**) MobileNetV2 classification results.

In Fig. 6 the histograms of the MSE are shown, overlapped with the error values obtained by^23^ in the same test sets. To be consistent with the comparison, all the networks have been quantized to 10 bits. The histograms for^23^ are derived from the figure in the paper and may not be very accurate.

All the MSE values lay in the range $[0,10 \times 10^{-4}]$. The results of the Pseudo-Softmax inference shows that this method is one order of magnitude better in approximation error than the method used in^23^.

#### Inputs quantization analysis

**Figure 7:**
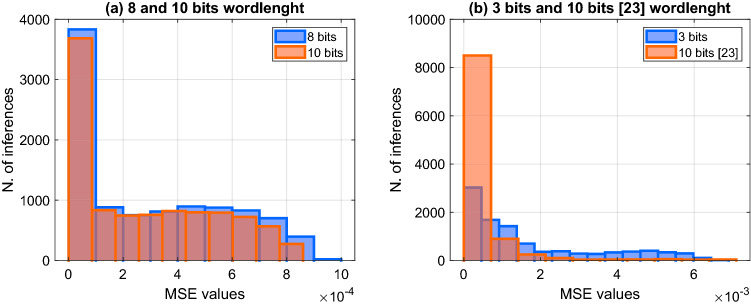
VGG-16 classification results for different quantizations: (**a**) 8 bits and 10 bits, (**b**) 3 bits and 10 bits of^23^.

As stated in the Introduction, typical hardware implementations of NNs are based on INT8 quantization. To see the impact of the NN quantization in the ImageNet test, the softmax MSE error histogram is evaluated while reducing the wordlenght of the inputs values $x_i$. In Fig. 7a the MSE values for 8 and 10 bits quantized VGG-16 networks are very similar. and therefore, Psuedo-Softmax architecture is quite insensitive for the quantization in that range of bits.

Similar results are obtained for the other tested networks.

By further reducing the input wordlenght, we obtain the minimum MSE achieved in^23^ ($10^{-3}$) for the 10 bits quantization when the inputs to our pseudo-softmax unit are 3 bits. The comparison of the MSE for the 10,000 patterns of the two methods applied to VGG-16 is illustrated in Fig. 7b. The histogram for^23^ is derived from the figure in the paper and may not be very accurate.

### Implementation results

We implemented the pseudo-softmax architecture by using a 90 nm 1.0 V CMOS standard-cell library. Since the standard-cell library is the same feature size than the one used in^23^, although the vendors may be different, the comparison of the implementation results is sufficiently fair. The synthesis was performed by using Synopsis Design Compiler. We considered 10 classes (N = 10) architectures.

**Figure 8:**
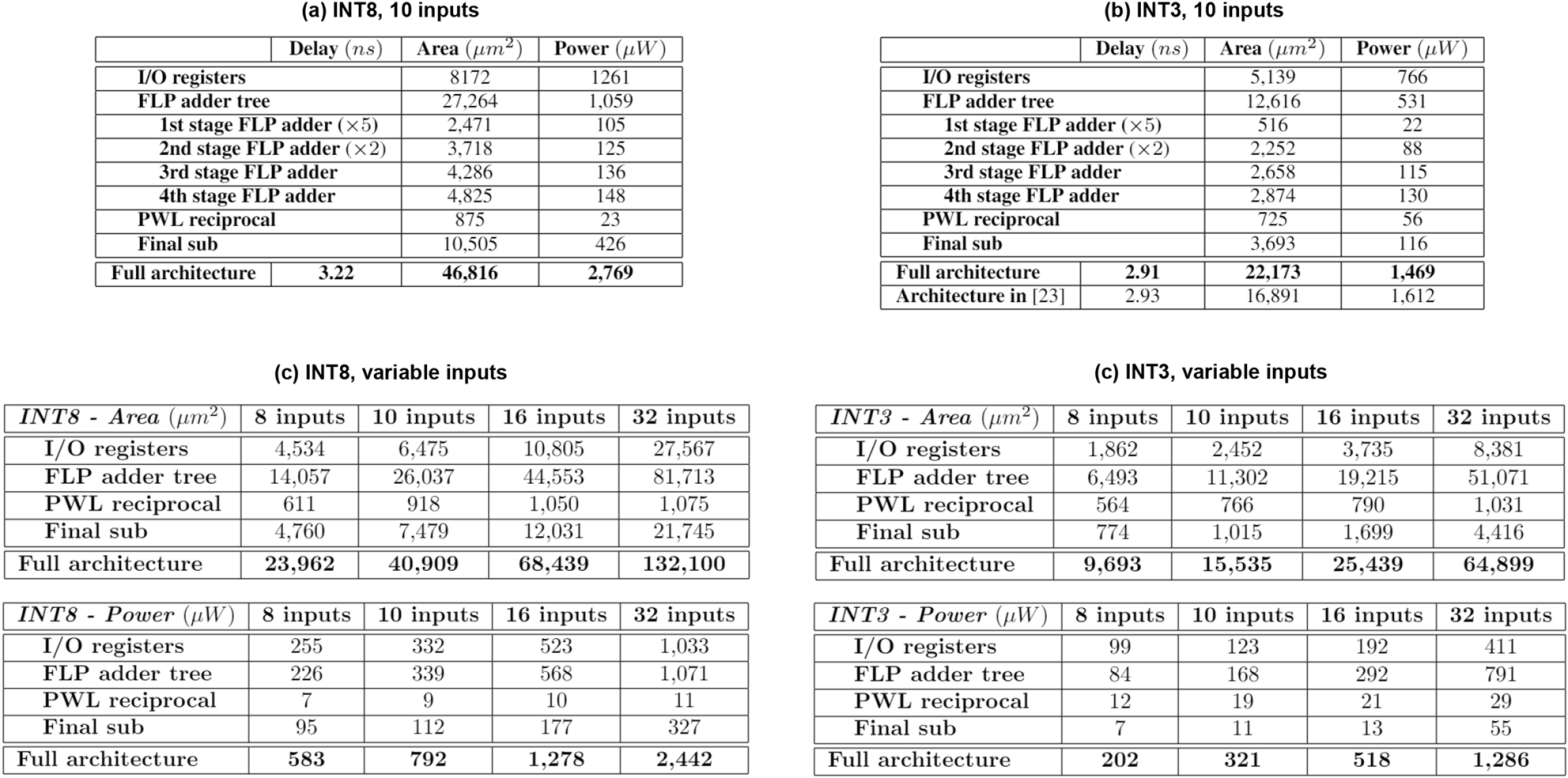
(**a**) Pseudo-softmax implementation results for a INT8, N = 10 classes architecture. (**b**) Pseudo-softmax implementation results for a 3 bit quantized, N = 10 classes architecture, and comparison with^23^. (**c**) Pseudo-softmax INT8 architectures implementation results for different number of inputs. (**d**) Pseudo-softmax INT3 architectures implementation results for different number of inputs.

The first implementation of the pseudo-softmax unit is for a INT8 input and N = 10 architecture. The results are reported in Fig. 8a. The input to output delay is 3.22 ns (the unit is not pipelined). The power dissipation is evaluated at the maximum operating frequency of 310 MHz.

Based on the result of the quantization analysis, the second implementation is a pseudo-softmax unit with 3-bit inputs. This unit gives a similar $\text {MSE} \propto 10^{-3}$ as the unit in^23^.

The result of the comparison are displayed in Fig. 8b. For “Architecture in^23^”, we rewrote the values from the paper for the fastest architecture identified as “Figure 2a”. The power dissipation was evaluated at 300 MHz.

By comparing the results in Fig. 8b, the delay is the same, the area of the pseudo-softmax is about 30% larger than the unit in^23^, and the power is not really comparable because we do not have any info on the clock frequency used to evaluate the power dissipation in^23^.

However, since the pseudo-softmax unit requires only 3-bit inputs for the same MSE, it is reasonable to assume that the neural network driving it can be quantized at a narrower bitwidth and be significantly smaller than a network producing 10-bit $p_i$s.

In Fig. 8c,d we provide the area and power dissipation for different INT8 and INT3 implementations, varying the number of inputs. We set the synthesis tool to a timing constraint of 100 MHz, which is the maximum achievable frequency of the larger architecture (INT8, 32 inputs). The power dissipations were evaluated considering this frequency.

Except for the PWL reciprocal block, the hardware resources are strictly related to the number of inputs and the quantization. Moreover, it can be observed how the area required for the I/O registers, the FLP adder tree, and the array of subtractors, doubles when we double the number of inputs.

## Discussion

In this paper, we proposed a pseudo-softmax approximation of the softmax function and its hardware architecture. The approximation error, measured by the MSE, is smaller than other softmax approximations recently presented.

Moreover, the pseudo-softmax function follows the property of probability distributions and its output values can be interpreted as probabilities.

Beside artificial NNs, the pseudo-softmax approximation method can be adapted to implement the Boltzmann action selection policy used in Reinforcement Learning.

The pseudo-softmax architecture has been implemented in VHDL and synthesized in standard-cells. The implementation results show that although the area of the pseudo-softmax unit is larger than the unit in^23^, its reduced inputs bitwidth can lead to an area reduction of the driving NN.

In a future extension of this work, the pseudo-softmax architecture could be rearranged to work with serial or mixed parallel–serial inputs. This would allow its hardware implementation in networks with a high number of output classes.
