# Author Correction: A pseudo-softmax function for hardware-based high speed image classification

**DOI:** 10.1038/s41598-021-97079-9

**Published:** 2021-09-01

**Authors:** Gian Carlo Cardarilli, Luca Di Nunzio, Rocco Fazzolari, Daniele Giardino, Alberto Nannarelli, Marco Re, Sergio Spanò

**Affiliations:** 1 Department of Electronic Engineering, University of Rome “Tor Vergata”, 00133 Rome, Italy; 2 Department of Applied Mathematics and Computer Science, Danmarks Tekniske Universitet, 2800 Kongens Lyngby, Denmark

Correction to: *Scientific Reports* 10.1038/s41598-021-94691-7, published online 28 July 2021

The original version of this Article contained an error in Figure 3 where panels (b) and (c) were incorrectly captured. The original Figure 3 and accompanying legend appear below.

**Figure 3:**
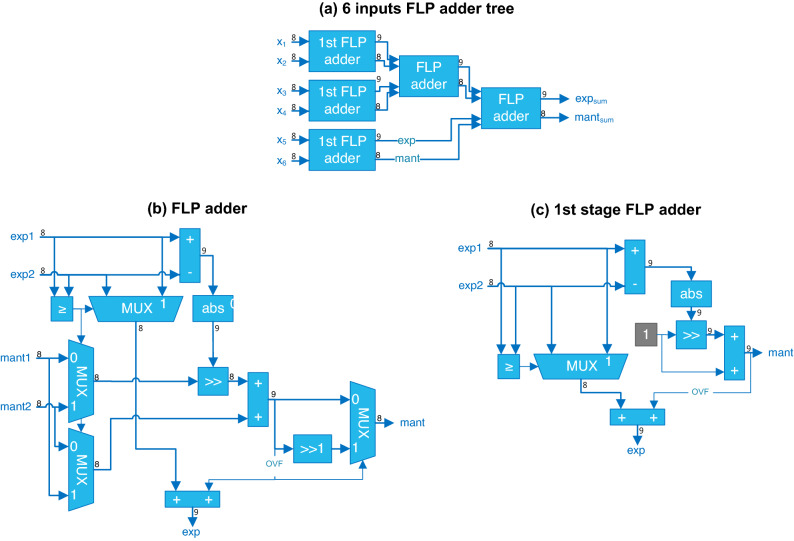
(**a**) Example of 6 8-bit inputs FLP adder tree. (**b**) Architecture of a FLP adder. (**c**) Architecture of the optimized FLP adder used in the first level of the tree.

The original Article has been corrected.

